# Safe administration of chemotherapy in mast cell activation syndrome

**DOI:** 10.1177/1078155220953879

**Published:** 2020-10-09

**Authors:** MP Lythgoe, J Krell, IA McNeish, L Tookman

**Affiliations:** Department of Surgery & Cancer, Imperial College Healthcare NHS Trust, Hammersmith Hospital, London, UK

**Keywords:** Mast Cell activation syndrome, endometrial cancer, carboplatin, carboplatin desensitization, paclitaxel

## Abstract

**Introduction:**

Mast Cell Activation Syndrome (MCAS) is an immunogenic disorder typically presenting with episodic multi-organ symptoms, caused by the inappropriate and aberrant release of mast cell mediators. Symptoms may be severe, including anaphylaxis and often occur in response to specific triggers which include many drugs and potentially chemotherapeutic agents. The administration of adjuvant chemotherapy and radiotherapy in endometrial cancer significantly reduces the risk of reoccurrence in patients with high risk disease. Currently there is no evidence or case reports to guide the safe administration of chemotherapy in MCAS patients.

**Case report:**

We present the case of a 59-year-old lady with stage 3 A grade 2 endometroid endometrial cancer who underwent successful surgical management. She then received 4 cycles of adjuvant chemotherapy in the form of carboplatin and paclitaxel. This case describes a staged approach to chemotherapy administration and the utilisation of a carboplatin desensitization regimen to reduce the risk of immediate and delayed hypersensitivity sequalae.

**Management & outcome:** Utilising an enhanced pre-medication strategy and a staged approach to chemotherapy administration, she was able to complete adjuvant treatment without any serious complications. At the date of censoring (May 2020) she has not shown any evidence of disease re-occurrence.

**Discussion & conclusion:** Administering chemotherapy to patients with any mast cell disorder remains challenging. We hope that this case may provide the framework for safer chemotherapy administration for any patients at high risk of serious hypersensitivity sequalae in endometrial cancer and beyond.

## Introduction

Mast cell activation disorders (MCAS) are a heterogeneous group of conditions associated with mast cell hyperreactivity and/or proliferation.^1^ Patients experience signs and symptoms attributable to inappropriate mast cell activation and pro-inflammatory mediator release. Symptoms range from mild, self-limiting allergic symptoms to potentially life-threatening recurrent anaphylaxis.

Mechanistically, MCAS can be classified as primary, secondary or idiopathic based on biochemical, molecular and clinical factors.^2^,^3^ In primary MCAS genetic abnormalities, such as mutations in the tyrosine kinase receptor *KIT,* results in a monoclonal proliferation of mast cells.^4^ Secondary MCAS occurs as an indirect result of another disease and/or inflammatory process, most commonly IgE-dependent allergic disease. Idiopathic MCAS is diagnosed when no primary or secondary causes are identified, despite extensive investigation. Many different drugs including chemotherapeutic agents (including carboplatin and paclitaxel) are known to be potent mast cell stimulators, therefore careful consideration is required before initiating any new procedure or treatment.^5^,^6^

Endometrial cancer (EC) is the fourth most common female cancer and the 8th leading cause of cancer-related deaths.^7^ Over 70% of endometrial cancers are detected at an early stage (stage 1 or 2) with the remainder being advanced disease with regional (stage 3) or distant (stage 4) metastasis.^8^ Surgery is the initial mainstay of management of EC. However, patients with advanced disease (stage 3 or 4) or those with high risk features (clear cell, serous adenocarcinoma or grade 3 invasive endometroid carcinoma) have a greater risk of disease re-occurrence with surgical management alone.^9^,^10^ Thus, adjuvant treatment to reduce the risk of re-occurrence and extend relapse-free survival is indicated. Chemotherapy and radiotherapy (pelvic and brachytherapy) are the two modalities of treatment which are given in the adjuvant setting.^11^,^12^ At the time of treatment (January 2018) the standard of care at our institution was a combination of chemotherapy (carboplatin and paclitaxel) followed by radiotherapy (pelvic and/or brachytherapy).

We present a case of how chemotherapy can be pragmatically administered safely to a patient with MCAS at high risk of serious allergic sequalae. There are currently no case reports to guide management in this situation and we hope this may provide a safe framework for other to follow in this unusual circumstance.

## Case report

A 59-year old lady presented to her primary care physician (September 2017) with a 4-week history of vaginal bleeding and weight loss. She was referred urgently to a tertiary gynaecology-oncology centre for further investigations. Initial evaluation consisted of blood tests, hysteroscopy and CT imaging of the chest, abdomen and pelvis. Her case was evaluated at the gynaecology oncology multi-disciplinary team (MDT) meeting and a provisional diagnosis of endometroid EC (likely stage 3) was agreed. It was recommended to proceed initially for surgical management.

The patient had a past medical history of hypertension, type 2 diabetes mellitus and most significantly, idiopathic mast cell activation disorder (MCAS) with a persistently raised tryptase. This condition initially presented 7 years ago with recurrent anaphylaxis. A plethora of different triggers have been established including foods (such as chicken, various nuts and seeds) and mast cell triggering drugs such as penicillin and macrolide antibiotics, experiencing both immediate and delayed reactions (up to 48 hours later). This condition was managed at a specialist allergy centre and she took a number of mast cell stabilising/allergy medications including sodium cromoglicate, fexofenadine, ketotifen, montelukast and cetirizine. She also took intermittent short courses of corticosteroid (two courses in past year) following severe reactions. However, despite these measures she continued to experience recurrent anaphylaxis with between two to four life-threatening episodes annually. Patch testing of potential triggers proved inconsistent and unreliable, failing to delineate any definitive triggers.

Recommended surgery consisted of a hysterectomy and salpingo-oophorectomy. Due to her diagnosis of MCAS her operation required meticulous planning, avoiding drugs with proven or theoretical high risk of mast cell activation such as muscle relaxants (e.g. atracurium), anaesthetic drugs (e.g. thiopental) and opioid analgesics (e.g. morphine). The operation proceeded and had no significant complications. She made an excellent recovery and was discharged five days after admission. Following surgery her case was re-discussed at the gynaecology oncology MDT with definitive histology from the operation. Her diagnosis was confirmed as a fully resected (R0 resection) stage IIIA grade 2 endometroid EC with lymphovascular space infiltration. Due to these high risk features she was referred to the oncology team for consideration of adjuvant chemotherapy and radiotherapy.

Currently guidelines for adjuvant treatment in EC recommend between four and six cycles of chemotherapy with carboplatin and paclitaxel followed by radiotherapy.^11^,^12^ However, in the context of administering chemotherapy in MCAS there are currently no guidelines or case reports to guide management. This patient was reviewed in the oncology clinic and these issues were explored. Due to her higher risk of reoccurrence she wished to attempt adjuvant chemotherapy to minimise the disease reoccurrence risk.

Liaising with her allergy team, consideration was given to patch test for both carboplatin and paclitaxel, however given the previous unreliability of this test, time scale and the lack of utility for predicting sensitivity to taxanes, this was not deemed appropriate. Taking into account her risk factors for hypersensitivity (MCAS, postmenopausal) it was agreed to proceed cautiously with a staged approach (Table 1) of 4 cycles every 3 weeks of adjuvant chemotherapy (carboplatin and paclitaxel).

**Table 1.** Schedule of Treatment (3-week interval between treatments).

	Drug	Route	Dose
Cycle 1			
Day -1: pre-medications (12hours before)	Dexamethasone	PO	8 mg
	Ranitidine	PO	150 mg
	Cetirizine	PO	10 mg
Day 1: pre-medications (30 minutes before)	Dexamethasone	IV	8 mg
	Ranitidine	IV	50 mg
	Ondansetron	IV	8 mg
	Chlorpheniramine	IV	10 mg
Day 1: chemotherapy	Carboplatin (AUC 5)*Via Desensitization regimen*	IV	560 mg
Cycle 2			
Day -1: pre-medications (12hours before)	Dexamethasone	PO	8 mg
	Ranitidine	PO	150 mg
	Cetirizine	PO	10 mg
Day 1: pre-medications (30 minutes before)	Dexamethasone	IV	8 mg
	Ranitidine	IV	50 mg
	Ondansetron	IV	8 mg
	Chlorpheniramine	IV	10 mg
Day 1: chemotherapy	Carboplatin (AUC 5) *Via Desensitization regimen*	IV	560 mg
Day 2: pre-medications (30 minutes before)	Dexamethasone	IV	20 mg
Day 2: chemotherapy	Paclitaxel (175 mg/m^2^)Via 6-hour infusion	IV	378 mg
Cycle 3			
Day -1: pre-medications (12 hours before)	Dexamethasone	PO	8 mg
	Ranitidine	PO	150 mg
	Cetirizine	PO	10 mg
Day 1: pre-medications (30 minutes before)	Dexamethasone	IV	8 mg
	Ranitidine	IV	50 mg
	Ondansetron	IV	8 mg
	Chlorpheniramine	IV	10 mg
Day 1: chemotherapy	Carboplatin (AUC 5)Via 1-hour infusion	IV	560 mg
Day 2: pre-medications (30 minutes before)	Dexamethasone	IV	20 mg
Day 2: chemotherapy	Paclitaxel (175 mg/m^2^)Via 6-hour infusion	IV	378 mg
Cycle 4			
Day -1: pre-medications (12hours before)	Dexamethasone	PO	20 mg
	Ranitidine	PO	150 mg
	Cetirizine	PO	10 mg
Day 1: pre-medications (30 minutes before)	Dexamethasone	IV	20 mg
	Ranitidine	IV	50 mg
	Ondansetron	IV	8 mg
	Chlorpheniramine	IV	10 mg
Day 1: chemotherapy	Carboplatin (AUC 5) Via 1-hour infusion	IV	560 mg
Day 2: pre-medications (30 minutes before)	Dexamethasone	IV	20 mg
Day 2: chemotherapy	Paclitaxel (175 mg/m^2^)Via 3-hour infusion	IV	378 mg

**Note**: Patient continued with regular medicines throughout treatment (sodium cromoglicate, fexofenadine, ketotifen, montelukast and cetirizine).

The first step to ensure accurate dosing of her chemotherapy (particularly carboplatin) was to estimate her renal function. Therefore, a glomerular filtration rate (GFR) analysis with ^51^Cr- ethylenediamine tetra-acetic acid (EDTA) was performed. Mild allergic phenomenon has been reported with administration of ^51^Cr-EDTA, however the incidence has not been quantified but is believed to be low.^13^ Therefore, as a precautionary measure she was pre-treated with an intravenous antihistamine (chlorpheniramine 10 mg) thirty minutes before proceeding. This investigation was completed satisfactorily without incident.

The preferred chemotherapy regimen is currently carboplatin and paclitaxel given every 3 weeks. No trials exist comparing the efficacy of either single agent in the adjuvant setting. However, when evaluating the single agent response rate in metastatic EC chemotherapy naïve patients and the hypersensitivity risk of each drug, it was agreed to initially commence with carboplatin, adding paclitaxel in later cycles if no allergic sequalae or other problems had been encountered.

Traditionally this chemotherapy regimen is given as an outpatient. However, due to her risk of hypersensitivity reactions and having previously experienced both immediate and late-onset severe hypersensitivity reactions to drugs, it was agreed she should receive chemotherapy as an inpatient. To minimise her risk of hypersensitivity sequalae she would also receive pre-medication enhancement drugs and carboplatin as part of a phased 4-step regimen (see Table 2). Phased carboplatin desensitization regimes have been developed to induce short-lived tolerance to facilitate administration in patients who initially developed significant carboplatin hypersensitivity and was deemed appropriate for use in this setting to enhance safety.^14^

**Table 2.** Carboplatin desensitization protocol.

	Concentration of infusion	Infusion rate
Infusion 1	0.1% of total carboplatin dose^a^	IV in 100 mls of glucose over 30 minutes
Infusion 2	1% of total carboplatin dose^a^	IV in 100 mls of glucose over 30 minutes
Infusion 3	10% of total carboplatin dose^a^	IV in 100 mls of glucose over 30 minutes
Infusion 4	Remainder of carboplatin dose^a^	IV in 100 mls of glucose over 1 hour

Note: Patients should receive pre-medication schedule as outlined in Table 1 (cycle 1).

^a^Carboplatin total dose is calculated based on Calvert formula: 5 x (Glomerular Filtration Rate* + 25).

*Glomerular Filtration Rate (GFR) as calculated by ^51^Cr-EDTA GFR scan (alternative would be Creatinine Clearance based on Cockcroft-Gault Equation).

The night before her chemotherapy she took oral corticosteroid, antihistamine and H_2_ receptor antagonist (in addition to her normal mast cell stabilising medications) before being admitted to hospital on the morning of planned treatment. On admission she had her bloods re-checked for suitability and a physician safety assessment to confirm fitness for treatment. She received pre-medication with intravenous corticosteroid, antihistamine, 5HT_3_ receptor antagonist and H_2_ receptor antagonist thirty minutes before chemotherapy. The patient’s total dose of carboplatin (560 mg) was calculated based on the Calvert formula (see Table 2) with an area-under the curve (AUC) of 5.^15^ This was prepared in four increasing concentrations of the total dose: 0.1%, 1%, 10% and standard infusion concentration following a predefined protocol.^15^,^16^ Each infusion was administered over 30 minutes, except for the highest concentration which was given over 1 hour. Prior to proceeding to the next concentration level, she was examined for signs of hypersensitivity. She completed the treatment without any complications and was observed overnight as an extra-precaution before being discharged.

Following successful treatment without any hypersensitivity sequalae she was reviewed in clinic and consideration given to the stage of her treatment. She reported some constitutional symptoms such as fatigue and dry skin, however overall tolerated the treatment well. After careful consideration it was agreed for her next treatment cycle to continue with the carboplatin desensitization regimen and if she continued to tolerate this without complication, to receive paclitaxel the following day. Paclitaxel has a higher risk of immediate hypersensitivity reaction, therefore was given at a slower infusion rate (normally 3 hours) of 6 hours. As per the standard protocol the pre-medication corticosteroid dose (dexamethasone 20 mg) was also increased. She attended for cycle 2, three weeks after her first and received her chemotherapy without incident and was discharged after completing the second infusion.

For the third cycle as she had tolerated both drugs without incident, it was agreed she should continue to receive the drugs as an inpatient on consecutive days. However, as she had not experienced any problems with carboplatin, she should receive this normally (over 1 hours), not via the desensitization protocol and the paclitaxel infusion at the normal rate the next day. This cycle was again completed without any significant adverse events.

For her final cycle she was given carboplatin and paclitaxel on consecutive days as an outpatient but continuing with the enhanced pre-medication. Unfortunately, on the second day during the paclitaxel infusion, she developed a minor hypersensitivity reaction (flushing, back pain). Her infusion was immediately stopped, and she was treated with intravenous hydrocortisone and chlorpheniramine. She responded well to this management and her symptoms stopped within 30 minutes and was able to complete the infusion at a slower (6 hours) infusion rate.

## Management & outcome

Following successful completion of 4 cycles of adjuvant chemotherapy she moved onto the next phase of her treatment. She completed adjuvant radiotherapy in the form of 45 Grey external beam radiotherapy (in 25 fractions) and 8 Grey high dose rate brachytherapy (in 2 fractions).

This was completed without incident. She has continued on regular follow-up since this time and at the time of censoring (May 2020) has had no evidence of disease re-occurrence.

## Discussion

Currently there are no recognised protocols to guide safe administration of chemotherapy in patients with MCAS. The combination of carboplatin and paclitaxel chemotherapy agents is an established regime for the adjuvant treatment of endometrial cancer in women with high risk disease.^12^ The hypersensitivity profiles of paclitaxel and carboplatin differ considerably. Paclitaxel is associated with a higher incidence of hypersensitivity (8-45%).^17^,^18^ Reactions typically occurs immediately (within 10 minutes) and only 1.3% are severe (grade 3 or 4 reaction).^17^ Carboplatin has a lower incidence of hypersensitivity; reactions become more frequent with cumulative exposure. In patients receiving up to 6 cycles of chemotherapy the reported incidence is <1%, making this ideal in the adjuvant treatment setting.^17^,^19^ However, beyond 6 cycles this rises to 27% and half are classed as moderate or severe reactions.^17^ Factors pre-disposing patients to hypersensitivity for both agents include any prior history of drug allergies or pre-existing hypersensitivity disorder (including mast cell disorders). Reactions in patients with pre-existing allergic conditions are characteristically more severe in nature and less responsive to pre-medication and treatment strategies. Recent evidence has also demonstrated than concomitant carboplatin and paclitaxel is associated with a higher hypersensitivity risk than alternative carboplatin combination regimens.^20^

Currently there are no trials evaluating use of single agent chemotherapy in the adjuvant setting for EC. However, information can be gleaned from single agent chemotherapy naïve metastatic EC trials. The most active single agent drugs are platinum agents, taxanes and anthracyclines all producing similar response rates of 20–30%.^21^ Therefore, when considering a treatment paradigm for this patient, evaluating the hypersensitivity safety profile of carboplatin for 4 cycles and the similar response rates of both drugs, carboplatin was selected as the initial drug of choice.

Desensitization is an allergological procedure primarily utilised to induce a state of temporary tolerance to facilitate essential drug administration. Drug desensitization regimens are typically used when no alternative drug is available and the benefits of such as treatment outweigh potential risks. In this case we utilized a carboplatin desensitization regimen to facilitate safer chemotherapy administration to a patient deemed to be at extremely high risk of developing both immediate and delayed life threatening allergic sequalae. This is the first reported use of this methodology and we hope this may be suitable in other high-risk cases. This approach did allow successful administration, however required significant additional healthcare resources (inpatient admission, special pharmacy chemotherapy preparation) to facilitate treatment.

Systemic mast cell activation diseases (such as systemic mastocytosis) have an associated increased risk of solid cancers including melanoma and non-melanoma skin cancers.^22^ In MCAS the cancer association risk is less well-defined, however registry studies have identified significantly increased prevalence of melanoma and cancers of the breast, ovary, cervix uteri, thyroid and lung.^23^ Taking account of the increasing utility of anti-cancer treatments including chemotherapy and the higher risk of malignancy in patient with mast cell disorders, this is likely to be a situation encountered more frequently in the future, therefore appropriate strategies are required.

## Conclusion

Administering chemotherapy in any setting to patients with a history of severe hypersensitivity reactions and anaphylaxis can be challenging. This case demonstrates a framework to allow cautious chemotherapy administration in patients at high risk of serious allergic sequalae. We have presented this case in the hope that this can be used in similar setting to facilitate patients with a history of hypersensitivity to receive chemotherapy safely and pragmatically.
